# Remarkable Case of Suicide, and Extraction of a Needle from the Substance of the Heart

**Published:** 1847-12

**Authors:** 


*Remarkable Case of Suicide, and Extraction of a Needle from the Substance of the Heart.*

NASHUA, N. H., SEPT., 1847.

[DEAR SIR :— A singular case occurred in Nashua, a few weeks since, which you may, perhaps, think of some interest. I will briefly and hastily state the facts, leaving you to make such disposition of them as you may think proper.]

On Sunday, the 16th of August last, one of the most desperate acts of self-destruction was committed by a young man, aged 23 years, in Nashua, N. H. The young man had been slightly indisposed for a day or two previous to the act, and confined to his room. He requested his father, who was sitting near him, to leave the room, as he wished to get some sleep. He left the room for a short time, and on returning, found his son deluged in blood, with his throat cut most shockingly. I was soon in attendance—found the patient nearly lifeless, with three extensive cuts across the neck. The cuts were through the hyoid, and between the thyroid and cricoid cartilages, severing entirely the larynx. On the left side, over and along the course of the fifth rib, there was an extensive cut down to the rib. During the hæmorrhage, the trachea had become nearly filled with blood, rendering his breathing extremely difficult. I turned him over upon the side, when, he quackled, and with a convulsive effort threw out a large quantity of blood from the trachea. I secured the bleeding vessels, dressed the wound, and left the house, with orders to give the patient brandy and water. After the lapse of two hours, or more, the messenger came again, saying that the patient had roused and wished to see me immediately. On my enteriug the patient's room, he said, "Doctor, I have got a darning-needle in my heart." I inquired how the needle came in his heart. His reply was, that he put the needle into his side previous to using the razor—that he feared the needle was not going to make sure work, &c. He placed his finger upon the spot where he said he put the needle, which was just between the fifth and sixth ribs. At this point there was a puncture in the skin, like the puncture of a pin or needle. He at this time had the appearance of great suffering—his pulse rapid and strong—his breathing extremely difficult—every breath attended with a screech. From his own statements, and the attending symptoms in the case, I was of the opinion that there was something in his side or heart, and that I should be justified in making an effort to extract it. I accordingly mades an incision between the fifth and sixth ribs, down to the intercostal muscles and made my dissection laterally, but could not find any trace of the needle. My next step was to cut down to the pleura, which I did by dissecting up the intercostal muscles. I now placed my finger on the pleura and pressed gently down, when I thought I felt a sharp point come in contact with my finger with every pnlse of the heart. I now made my third incision through the pleura. It was now that I had a sight of the needle. By dilating the wonnd with the aid of retractors, I could distinctly see the heart act with the needle in it. With the aid of a pair of forceps, I extracted the needle, and it was followed with a forcible stream of blood. The patient soon became more quiet, breathing less difficult; pulse less frequent; slept some during the night. Second day, has no pain; breathing easy; pulse 90; sleeps well; takes nourishment with much difficulty, on account of the division of the œsophagus. He continued to improve daily, up to the sixth day, when he was attacked with pleuritic pains, inability to swallow, and died on the eighth day a ter the needle was taken from the heart.

*Post-mortem Appearance.*—Pleura slightly inflamed around the wound. On the inner surface of the pericardium there was a puncture, resembling a leech-bite, where the needle entered. The pericardium contained no blood, and the heart appeared natural. On opening into the left ventricle, we found where the needle entered this cavity. There was a small membranous sac, about as large as a pea, formed in the left ventricle, which contained pus. Nature, it seems, had set up a process by which to protect herself, by throwing around the needle this adventitious membrane.

I am, Sir, very respectfully, your obt. servant,

[*N. Y. Annalist.*] J. G. GRAVES.

